# Sincronia Ventricular na Estimulação Cardíaca Parahissiana: Alternativa por Ativação Cardíaca Fisiológica (Estimulação Indireta do Feixe de His)?

**DOI:** 10.36660/abc.20201233

**Published:** 2021-12-20

**Authors:** Andres Di Leoni Ferrari, Guilherme Ferreira Gazzoni, Luis Manuel Ley Domingues, Jessica Caroline Feltrin Willes, Gustavo Chiari Cabral, Flavio Vinicius Costa Ferreira, Laura Orlandini Lodi, Gustavo Reis

**Affiliations:** 1 Hospital São Lucas Pontifícia Universidade Católica do Rio Grande do Sul Porto Alegre RS Brasil Serviço de Cardiologia. Hospital São Lucas da Pontifícia Universidade Católica do Rio Grande do Sul (PUCRS), Porto Alegre, RS – Brasil; 2 Universidad Popular Autonoma del Estado de Puebla Facultad de Medicina Puebla México Universidad Popular Autonoma del Estado de Puebla - Facultad de Medicina, Puebla – México; 3 Eletrofisiologia Londrina Londrina PR Brasil Eletrofisiologia Londrina, Londrina, PR – Brasil

**Keywords:** Marca-Passo Artificial, Estimulação Cardíaca Artificial, Terapia por Estimulação Elétrica

## Abstract

**Fundamento:**

A estimulação cardíaca artificial (ECA) por captura direta ou indireta do feixe de His resulta em contração ventricular sincrônica (ECA fisiológica).

**Objetivos:**

Comparar sincronia cardíaca, características técnicas e resultados de parâmetros eletrônicos entre duas técnicas de ECA indireta do feixe de His: a não seletiva e a parahissiana.

**Métodos:**

Intervenção experimental (novembro de 2019 a abril de 2020) com implante de marca-passo definitivo (MPd) DDD em pacientes com fração de ejeção ventricular esquerda > 35%. Foram comparadas a sincronia cardíaca resultante mediante algoritmo de análise eletrocardiográfica da variância espacial do QRS e as características técnicas associadas a cada método entre ECA hissiana não seletiva (DDD-His) e parahissiana (DDD-Var).

**Resultados:**

De 51 pacientes (28 homens), 34 (66,7%) foram alocados no grupo DDD-Var e 17 (33,3%), no grupo DDD-His, com idade média de 74 e 79 anos, respectivamente. No grupo DDD-Var, a análise da variância espacial do QRS (índice de sincronia ventricular) mostrou melhora após o implante de MPd (p < 0,001). Ao ECG pós-implante, 91,2% dos pacientes do grupo DDD-Var mostraram padrão fisiológico de ECA, comprovando ativação similar à do DDD-His (88,2%; p = 0,999). O eixo do QRS estimulado também foi similar (fisiológico) para ambos os grupos. A mediana do tempo de fluoroscopia do implante foi de 7 minutos no grupo DDD-Var e de 21 minutos no DDD-His (p < 0,001), favorecendo a técnica parahissiana. A duração média do QRS aumentou nos pacientes do DDD-Var (114,7 ms pré-MPd e 128,2 ms pós-implante, p = 0,044). A detecção da onda R foi de 11,2 mV no grupo DDD-Var e de 6,0 mV no DDD-His (p = 0,001).

**Conclusão:**

A ECA parahissiana comprova recrutamento indireto do feixe de His, mostrando-se uma estratégia eficaz e comparável à ECA fisiológica ao resultar em contração ventricular sincrônica similar à obtida por captura hissiana não seletiva.

## Introdução

A evolução da estimulação cardíaca artificial (ECA) demonstrou que a condução do impulso por meio da ativação muscular não fisiológica do ventrículo direito (VD), principalmente a estimulação apical (ECA “convencional”), está associada a efeitos cardíacos deletérios e repercussões negativas.^1^ A ECA convencional soluciona o problema elétrico e hemodinâmico ao restaurar a frequência cardíaca, porém causa alterações eletromecânicas decorrentes da dissincronia cardíaca.^5^ A dissincronia se manifesta eletricamente (QRS alargado com padrão de bloqueio de ramo esquerdo) e mecanicamente (remodelamento cardíaco, regurgitação mitral e disfunção sistólica).^4,5^

Vários estudos ratificaram a viabilidade e os resultados clínicos positivos da estimulação direta do feixe de His (*direct His-bundle pacing*, DHBP) quando comparada à ECA convencional.^6^ Atualmente, podemos considerar a indicação da DHBP para quase todos os distúrbios da condução cardíaca. A padronização dessa técnica, porém, é desafiadora. Alguns critérios ainda precisam ser refinados, tais como diferenças clínicas, se presentes, entre estimulação seletiva (*selective His-bundle pacing*, S-HBP) e não seletiva (*nonselective His-bundle pacing*, NS-HBP)^9^do His; os maiores limiares de captura que resultam em desgaste acelerado da bateria do gerador; e os recursos adicionais (bainhas e eletrodos específicos) necessários para o posicionamento do eletrodo ventricular em contato com o feixe de His.^10,11^ Além disso, observa-se uma curva de aprendizado trabalhosa, com procedimentos mais longos, taxas de sucesso entre 60 e 90% e, em parte dos casos, dificuldades e complicações quanto à programação do dispositivo.^10,12^

A estimulação parahissiana (*paraHisian pacing*, PHP), por sua vez, apresenta uma curva de aprendizado mais rápida e de menor custo em termos de materiais, mostrando-se capaz de preservar, de modo similar, a sincronia da despolarização ventricular.^12,13^ A técnica consiste no posicionamento do eletrodo na região mais proximal do septo interventricular (IV) do VD, adjacente ao sistema de condução. Mais reprodutível, essa técnica é uma alternativa promissora para o conceito de estimulação cardíaca fisiológica ao indiretamente e rapidamente recrutar o sistema His-Purkinje, assemelhando-se à NS-HBP.^12^ O objetivo deste estudo é analisar comparativamente a sincronia cardíaca obtida através de duas técnicas que envolvem uma abordagem indireta do sistema de condução para ECA fisiológica: a NS-HBP e a PHP.

## Metodologia

Trata-se de um estudo de intervenção experimental realizado na Unidade de Estimulação Cardíaca e Ambulatório de Marca-Passos do Hospital São Lucas, da Pontifícia Universidade Católica do Rio Grande do Sul (PUCRS), em Porto Alegre, Brasil. Foram selecionados pacientes submetidos a implante de marca-passo definitivo (MPd) de dupla câmara DDD conforme as diretrizes vigentes,^14^ que possuíam fração de ejeção do ventrículo esquerdo (FEVE) preservada (> 50%) ou intermediária (36 a 49%).^15^ Todos os procedimentos de implante foram realizados pelo mesmo operador principal (ADLF). Todos os participantes assinaram o termo de consentimento livre e esclarecido. Excluíram-se pacientes com indicação de implante de cardiodesfibrilador, cardiodesfibrilador ou MPd multissítio ou MPd de câmara única e aqueles com dados incompletos para análise.

Os pacientes foram divididos em dois grupos: DDD-Var (implante de eletrodo VD parahissiano – PHP) e DDD-His (eletrodo VD em posição comprovada de captura do feixe de His não seletiva – NS-HBP), sendo o último guiado por mapeamento eletrofisiológico convencional.

**Figura 1:**
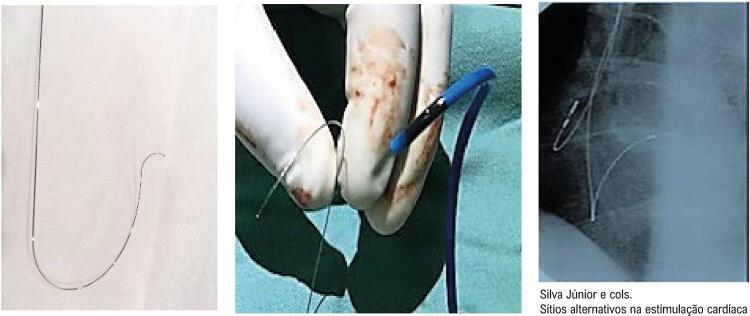
– Esquerda: estilete guia manualmente moldado para direcionamento e implante de eletrodo do ventrículo direito (VD) no 1/3 proximal do septo interventricular para a estimulação parahissiana. Centro: Operador (ADLF) mostrando comparação do formato obtido pela moldagem da guia com a curvatura de uma das bainhas pré-moldada disponível no Brasil (C315His Medtronic™). Direita: fluoroscopia oblíqua esquerda mostrando a posição final do eletrodo do VD. Nota-se angulação da ponta, perpendicular à coluna. Adaptado de Ortega et al. e Júnior et al.^12,19^

A técnica de posicionamento do eletrodo do VD em posição proximal do septo IV para PHP seguiu metodologia previamente descrita.^5,16^ Brevemente, o eletrodo ventricular (cabos bipolares convencionais, de fixação ativa, de todos os fabricantes do mercado) foi montado em estilete guia manualmente moldado com curvatura ampla no terço distal, seguido de curvatura mais acentuada na porção final, com direcionamento posterior (Figura 1).^5,12^

Guiado por anatomia radiológica, em projeção póstero-anterior, o eletrodo foi avançado para a artéria pulmonar e, com o fio guia totalmente inserido, foi tracionado para a via de saída do VD. Nessa projeção, o septo IV é dividido em três zonas:^19^ a superior (1/3 cranial do VD, entre o abaulamento da artéria pulmonar e o teto da valva tricúspide), a intermediária e a inferior (1/3 inferior do septo do VD). Em seguida, o posicionamento septal foi confirmado por incidência radioscópica em posição oblíqua anterior esquerda (entre 30 e 45 graus). Nessa incidência, o eletrodo está apontando perpendicularmente à coluna, em uma direção oposta à parede livre do VD^12,19,20^(Figura 1).

Para confirmar a PHP (grupo DDD-Var), buscou-se o complexo QRS estimulado mais estreito (até 130 ms e sempre < 150 ms) através do mapeamento do septo IV pelo eletrodo ventricular^21^ previamente à liberação do *screw-in.* Simultaneamente, em tempo real (intraoperatório), sob estimulação VVI decrementando desde amplitude = 5 V e largura de pulso = 1 ms, o sistema de análise da variância espacial do QRS – dispositivo Synchromax® (Exo S.A. Argentina) – mostrava o índice imediato de sincronia (IimeS). O sítio de PHP com o melhor índice era escolhido para a fixação definitiva do cabo do VD.

O IimeS resulta do processamento gráfico e matemático do sinal promediado da variância cruzada das derivações DII (septo IV direito) e V6 (parede lateral do VE). Para essa análise, o dispositivo Synchromax® utiliza a medida do fluxo de corrente elétrica (volume e sentido) e a análise de concordância das deflexões intrinsecoides dos QRS (Figura 2A).^12,22,23^ Valores de IimeS < 0,40 são considerados normais (sincrônico); valores de IimeS > 0,41 e < 0,69 são considerados dissincronia moderada; e valores de IimeS > 0,7 são considerados dissincronia severa (Figura 2B).^11,19,21^

**Figura 2A:**
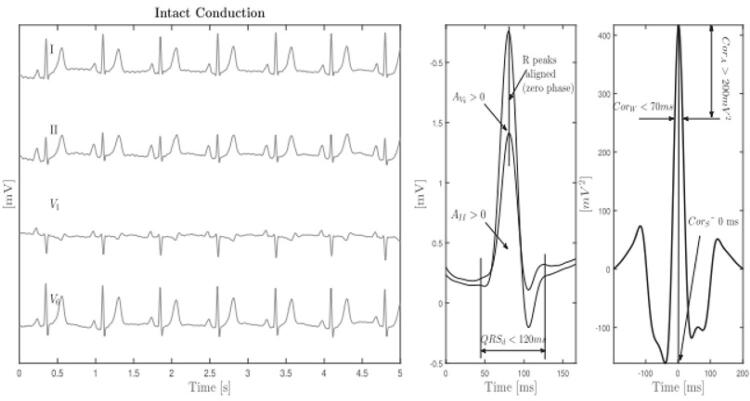
– Correlação da variância espacial do QRS e valores normais para um paciente com condução intraventricular intacta (derivações II e V_6_). Esquerda: traçados de eletrocardiograma. Centro: segmentos QRS sobrepostos (II^qrs^ e V_6_^qrs^). Direita: análise de correlação cruzada das derivações II e V_6_. Os picos dos complexos QRS coincidem, e o sinal de correlação cruzada mostra o seu máximo no tempo 0 (Cor_S_ = 0). Cor_S_: deslocamento de correlação cruzada (ms); Cor_W_: largura de correlação cruzada (ms); Cor_A_: amplitude de correlação cruzada (mV); A_II_: área sob a derivação II; AV_6_: área sob a derivação V_6_. Adaptado de Bonomini et al.^22^

**Figura 2B:**
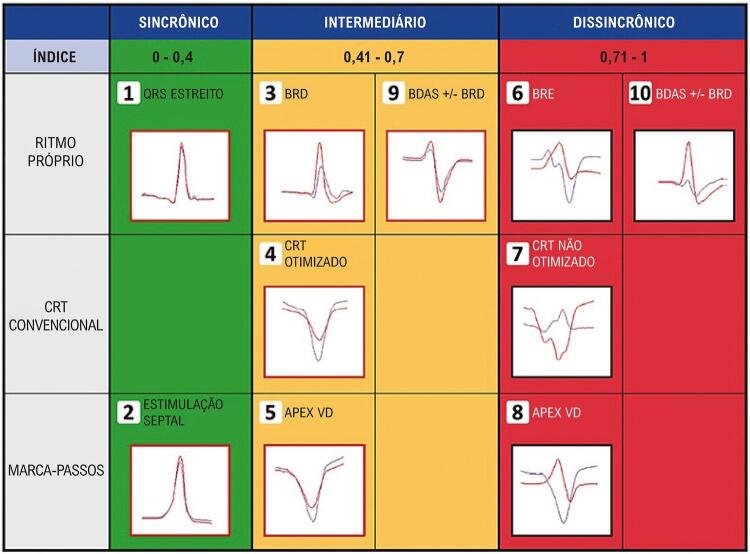
– Curvas com diferentes morfologias obtidas com o Synchromax^TM^ de acordo com o índice imediato de sincronia obtido em tempo real a partir do local de estimulação cardíaca artificial. Traços azuis: análise de variância do QRS da derivação II. Traços vermelhos: análise da variância espacial do QRS da derivação V_6._ BRD: Bloqueio de ramo direito; BRE: Bloqueio de ramo esquerdo; TRC: Terapia de Ressincronização Cardíaca; BDAS: Bloqueio Divisional ânterossuperior esquerdo; VD: ventrículo direito.

A abordagem e a captura do feixe de His (grupo DDD-His) foram feitas através de duas vias. Uma através da introdução femoral de cateter quadripolar para mapeamento eletrofisiológico e registro do potencial elétrico de His. A outra ocorreu através da introdução pela veia cefálica ou subclávia de bainhas defectíveis (C315-His, Medtronic, EUA) para o posicionamento do eletrodo de fixação ativa *lumenless* SelectSecure 3830 (Medtronic, EUA) em topografia de His, indicada pelo cateter de eletrofisiologia. Em seguida, confirmou-se a captura do feixe de His seletiva (S-HBP) ou não seletiva (NS-HBP).^24^ Simultaneamente e de forma não invasiva, durante o intraoperatorio, mediante estimulação VVI e decrementando a partir de energia de 5 V de amplitude x 1 ms de largura de pulso, realizou-se análise de variância espacial do QRS mediante dispositivo Synchromax® (Exo S.A. Argentina) determinando em tempo real o IimeS, sob as mesmas características metodológicas descritas acima na estimulação PHP. Os melhores valores da NS-HBP foram selecionados para estudo.

Além do IimeS (Synchromax®), foram registrados, para ambos os grupos, o tempo de fluoroscopia (Rx) dedicado ao procedimento e eletrocardiograma (ECG) de superfície intraprocedimento e previamente à alta. A ativação endocárdica local do VD confirmou-se através dos seguintes parâmetros eletrônicos: medição da amplitude da onda R, teste de limiar de captura unipolar e bipolar por estimulação decremental em modo VVI e impedância unipolar e bipolar. Os melhores valores foram selecionados para análise.

A análise dos ECG era “cegada” ao tipo de metodologia e às características (pré e pós) do implante e do paciente. Para a determinação do eixo elétrico dos QRS do ECG pós-implante, considerou-se fisiológica a presença de ativação ventricular da direita para esquerda (QRS positivo nas derivações D1 e AVL) e de cima para baixo (QRS positivo em DII, DIII e AVF), assim como transição (onda R ≥ S) até V3-V4 nas derivações precordiais.^25^ A presença de todos os três critérios categoriza um eixo fisiológico, a presença de dois critérios categoriza um eixo possivelmente fisiológico e a observação de somente um ou nenhum critério categoriza o eixo cardíaco como não fisiológico.

**Figura 3:**
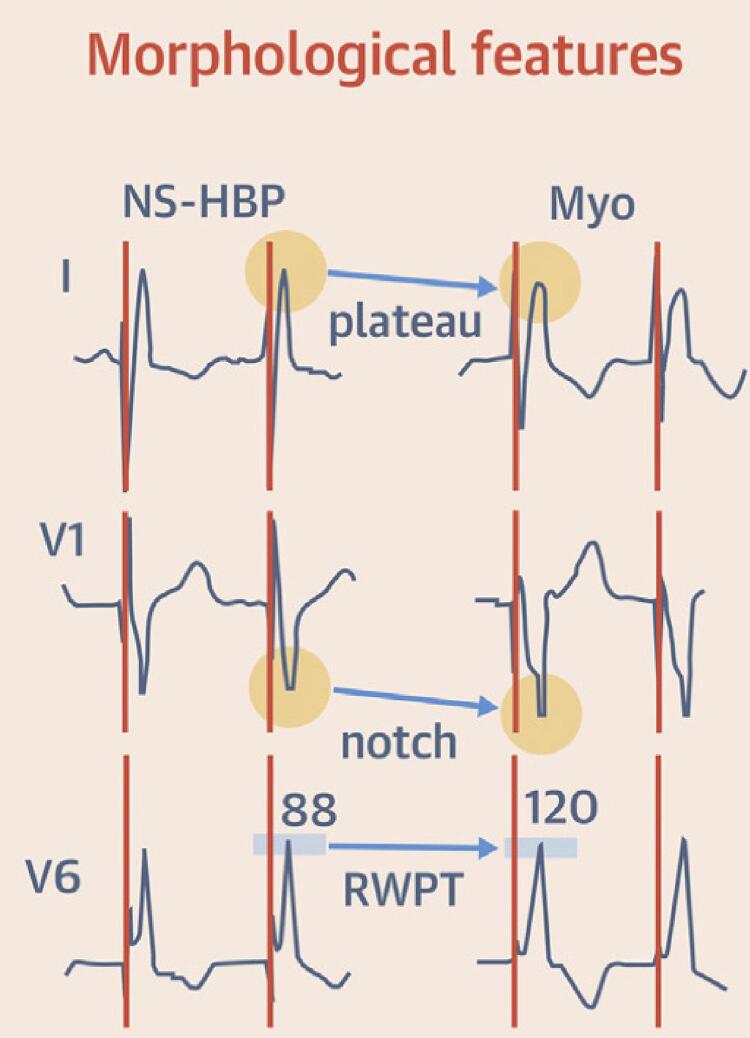
– Modelo eletrocardiográfico proposto por Burri et al.^26^ pela combinação de a) ausência de plateau em D1; b) ausência de entalhe (notch) na derivação V1; c) duração da ativação elétrica até o pico da onda R (RWPT) em V6 < 100 ms.^28–30^ Os três parâmetros presentes (a, b, c) indicam estimulação cardíaca artificial (ECA) fisiológica semelhante à NS-HBP e descartam ativação ventricular puramente miocárdica. A presença de dois critérios determina ECA provavelmente fisiológica, enquanto a presença de apenas um critério indica ECA provavelmente não fisiológica. A ausência de todos os elementos denota captura puramente do miocárdio inespecífico (ECA miocárdica).^28^

No grupo DDD-Var, para confirmar PHP sincrônica semelhante à NS-HBP e descartar estimulação muscular ventricular, utilizamos o modelo eletrocardiográfico proposto por Burri et al (Figura 3) verificando-se a combinação de ausência de *plateau* em D1, ausência de entalhe na derivação V1 e duração da ativação elétrica até o pico da onda R (RWPT) em V6 < 100 ms.^26^ A presença dos três parâmetros indica ECA fisiológica semelhante à NS-HBP e descarta ativação ventricular puramente miocárdica. A presença de dois desses critérios indica ECA provavelmente fisiológica, enquanto a presença de apenas um critério indica ECA provavelmente não fisiológica. Quando todos os elementos estavam ausentes, considerou-se apenas captura do miocárdio (ECA miocárdica).

Para todos os pacientes, apenas na análise qualitativa, acompanhou-se a evolução clínica aguda (até a alta hospitalar) com interesse em complicações de causa cardiovascular, especialmente as relacionadas ao implante de MPd.

## Análise estatística

As variáveis categóricas foram analisadas pelo teste exato de Fisher ou teste do qui-quadrado com correção de Yates, conforme distribuição das frequências nas diferentes categorias, e foram descritas por frequências e percentuais. A análise comparativa entre as variáveis categóricas, antes e depois do procedimento, foram associadas pelo teste de McNemar. As variáveis quantitativas com distribuição simétrica foram comparadas pelo teste *t* de Student para amostras independentes entre grupos; dentro dos grupos, foram comparadas pelo teste *t* de Student para amostras emparelhadas. As variáveis com distribuição assimétrica foram comparadas pelo teste de Mann-Whitney entre os grupos e pelo teste de Wilcoxon dentro dos grupos. A simetria das variáveis quantitativas foi analisada pelo teste de Kolmogorov-Smirnov e descrita pela média e pelo desvio padrão; em caso de distribuição assimétrica, foi descrita pela mediana e pelos valores mínimo e máximo. Foi considerado um nível de significância de 5% para as comparações estabelecidas. O *software* SPSS, versão 20.0, foi utilizado para a análise estatística.

## Resultados

No período de novembro de 2019 a abril de 2020, foram incluídos 51 pacientes no estudo, dos quais 34 (66.7%) foram alocados no grupo DDD-Var e 17 (33.3%), no grupo DDD-His. A idade média foi de 74 anos para o primeiro grupo e de 79 anos para o segundo, sendo a maioria homens (n = 28). A etiologia mais prevalente para o implante de MPd foi o bloqueio atrioventricular total no grupo DDD-Var e a disfunção do nó sinusal (DNS) no grupo DDD-His. A FEVE era preservada (> 50%) em 40 pacientes (78.4%) e intermediária (36 a 49%) em 11 (21.6%) pacientes. A comparação dos grupos é apresentada na Tabela 1.

**Tabela 1:** – Comparação das características dos grupos

	DDD-Var n = 34	DDD-His n = 17	Valor de p
Sexo masculino; n (%)	21 (61,8)	7 (41,2)	0,274
Idade em anos; média ± DP	74,0±8,9	79,0±7,9	0,063
**Doença de base; n (%)**			**0,004**
BAVT	17 (50,0)^a^	3 (17,6)^b^	
BAV 2º grau	9 (26,5)^a^	3 (17,6)^a^	
DNS	8 (23,5)^a^	11 (64,7)^b^	
Fração de ejeção preservada (> 50%); n (%)	27 (90,0)	13 (86,7)	0,999

*BAV: bloqueio atrioventricular; BAVT: bloqueio atrioventricular total; DNS: disfunção do nó sinusal; DP: desvio padrão. Variáveis categóricas associadas pelo teste exato de Fisher ou teste do qui-quadrado com correção de Yates. Variáveis quantitativas associadas pelo teste t de Student para amostras independentes. ^a,b^: letras diferentes indicam percentuais estatisticamente diferentes.*

### Sincronia cardíaca

Na amostra total, a análise da variância espacial do QRS (Synchromax®) mostrou significância estatística (p < 0,001) do IimeS na comparação do pré e do pós-operatório. Dos 20 pacientes sincrônicos no pré-operatório, 19 (95,0%) permaneceram sincrônicos no pós-operatório. Dos 31 indivíduos restantes, cinco (9.8%) eram intermediários (IimeS = 0,41-0,69), e 26 (51%) eram não sincrônicos (IimeS > 0,7). Após o implante, 30 (96,8%) tornaram-se sincrônicos, e apenas um (3.2%) permaneceu intermediário.

Detalhadamente, dentro do grupo DDD-Var, também houve variação significativa na categoria de IimeS (p < 0,001) entre o pré e o pós-implante. De 26 pacientes não sincrônicos, 25 (96,2%) tornaram-se sincrônicos e apenas um (3,8%), intermediário. Ainda, dos oito pacientes sincrônicos no pré-operatório, todos permaneceram sincrônicos pela medição do IimeS no pós-operatório. No grupo DDD-His, dos 12 indivíduos sincrônicos, 11 (91,7%) permaneceram sincrônicos, tendo apenas um paciente sido catalogado como não sincrônico no pós-operatório. Dos cinco pacientes restantes (não sincrônicos ou intermediários), todos (100%) foram convertidos para sincrônicos no pós-operatório (Tabela 2).

**Tabela 2:** – Comparação dos resultados pré e pós-implante do marca-passo definitivo

	DDD-Var	DDD-His	Valor de p
**Índice de sincronia cardíaca**	n = 34	n = 17	
Pré-implante^*^; n (%)			0,001
Sincrônicos	8 (23,5)^a^	12 (70,6)^b^	
Intermediários	3 (8,8)^a^	2 (11,8)^a^	
Não sincrônicos	23 (67,6)^a^	3 (17,6)^b^	
**Pós-implante; n (%)**			0,560
Sincrônicos	33 (97,1)	16 (94,1)	
Intermediários	1 (2,9)	-	
Não sincrônicos	-	1 (5,9)	
**Valor do IimeS**			
Pré-implante^*^; mediana (mínimo-máximo)	1,00 (0,12-1,00)	0,21 (0,06-1,00)	**0,001**
Pós-implante; mediana (mínimo-máximo)	0,18 (0,11-0,70)	0,18 (0,11-0,72)	0,461
% variação^†^; mediana (mínimo-máximo)	-74 (-89-192)	0 (-77-243)	**< 0,001**
			
ECG pós-implante	n = 34	n = 17	
Eixo; n (%)			0,074
Fisiológico	26 (76,5)	8 (47,1)	
Possivelmente fisiológico	8 (23,5)	9 (52,9)	
ECA	n = 34	n = 17	
Categoria (%)			0,915
Fisiológica	15 (44,1)	6 (35,3)	
Provavelmente fisiológica	16 (47,1)	9 (52,9)	
Provavelmente não fisiológica	3 (8,8)	2 (11,8)	
			
**Critério ausente de ECA fisiológica, n (%)**	n = 19	n = 11	
RWPT ≥ 100 ms	5 (26,3)	6 (54,5)	0,238
*Plateau* em D1	12 (63,2)	4 (36,4)	0,299
*Notch* em V1	5 (27,8)	3 (27,3)	0,999

Sincrônicos: índice de sincronia cardíaca (IimeS) ≤ 0,40; intermediários: IimeS = 0,41-0,70; não sincrônicos: IimeS ≥ 0,71. Variáveis categóricas associadas pelo teste exato de Fisher ou teste do qui-quadrado com correção de Yates. Variáveis quantitativas com distribuição assimétrica associadas pelo teste de Mann-Whitney. ^†^% variação = ([valor pós/pré valor pré]/valor pré)*100. ^a,b^: letras diferentes indicam percentuais estatisticamente diferentes. ECA: estimulação cardíaca artificial; ECG: eletrocardiograma; RWPT: pico da onda R. tempo de ativação até pico da onda R do QRS (ms). No “Critério ausente de ECA Fisiológica” um eventual paciente poderia mostrar mais de um critério.

**Figura 4:**
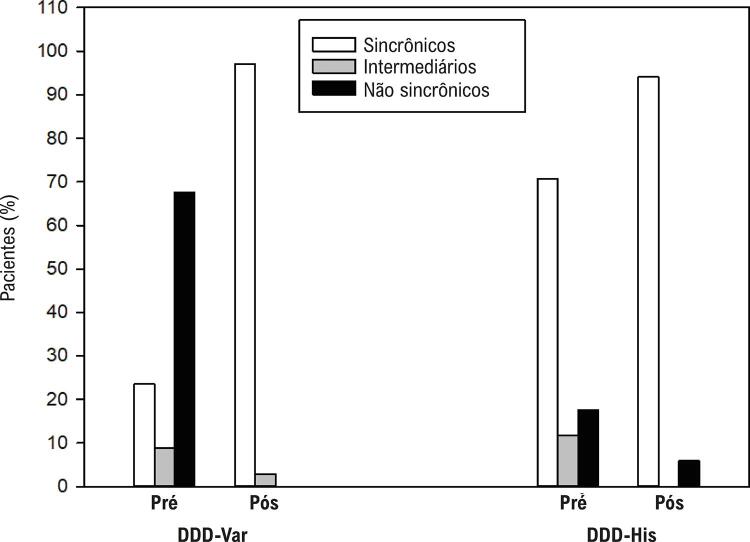
– Frequência de distribuição das categorias de sincronia cardíaca pré e pós-operatória entre os grupos.

**Figura 5:**
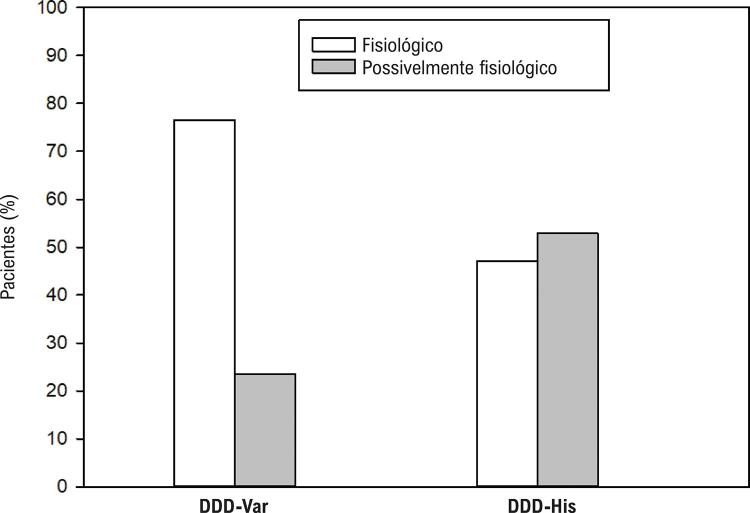
– Comparação do índice imediato de sincronia elétrica cardíaca entre os grupos.

A Tabela 2 também descreve as diferenças estatisticamente significativas entre os grupos no pré-operatório. O DDD-Var aglomerava mais pacientes não sincrônicos (67,6% *versus* 17,6% no DDD-His) e menos pacientes sincrônicos (23,5% *versus* 70,6% no DDD-His). No pós-operatório, os grupos se assemelharam, uma vez que foi obtida sincronia em ambos os grupos (97,1% no grupo DDD-Var e 94,1% no DDD-His; p = 0,560) (Figura 4). Os valores do IimeS também foram significativamente diferentes entre os grupos no pré-operatório (1,00 no DDD-Var e 0,21 no DDD-His, p = 0,001) e similares no pós-operatório (0,18 no DDD-Var e 0,18 no DDD-His, p = 0,461) (Figura 5), o que confirma a obtenção de ECA fisiológica com ambos os métodos, assemelhando a PHP à NS-HBP. O grupo DDD-Var teve redução mediana de 74% do IimeS (*versus* redução mediana de 0% no grupo DDD-His, p < 0,001), sinalizando a magnitude da correção da sincronia. Analisando cada grupo separadamente e comparando os dados de sincronia entre o pré e o pós-operatório, o DDD-Var demonstrou variação significativa (de 1,00 no pré para 0,18 no pós; p < 0,001) e, conforme o esperado, não houve diferença significativa de variação no DDD-His (mediana de 0,21 no pré para 0,18 no pós-operatório; p = 0,453).

### Eixo elétrico fisiológico

**Figura 6:**
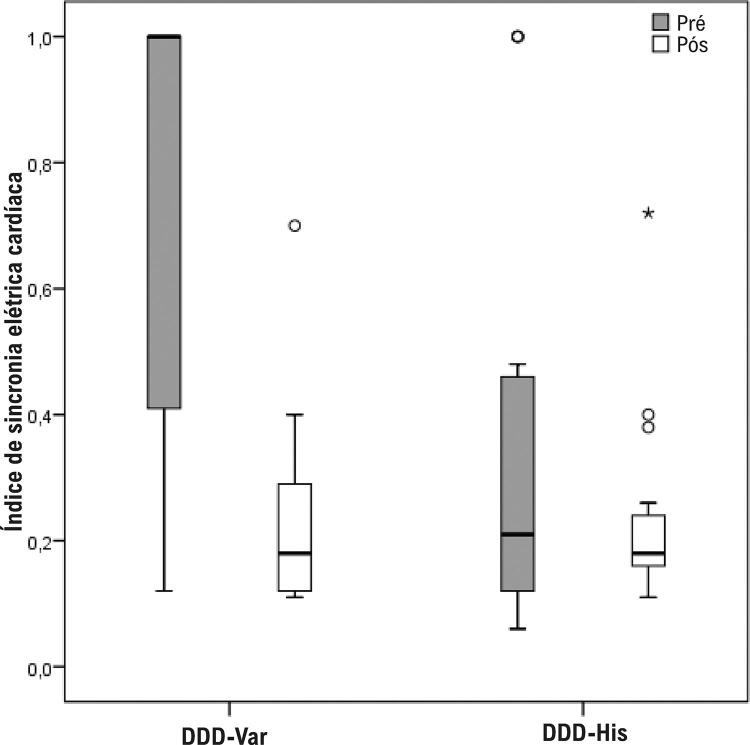
– Gráfico comparativo do eixo do eletrocardiograma entre os grupos.

A Figura 6 demonstra que o eixo elétrico do QRS pós-implante é similar para ambos os grupos (p = 0,074). Corroborando a aproximação entre os métodos quanto ao recrutamento do sistema de condução de His-Purkinje, não houve diferença (p = 0,915) na comparação do eixo resultante como possivelmente fisiológico (47,1% no DDD-Var e 52,9% no DDD-His) ou fisiológico (44,1% no DDD-Var e 35,3% no DDD-His).

### Estimulação cardíaca fisiológica: critérios de captura do sistema de condução

**Figura 7:**
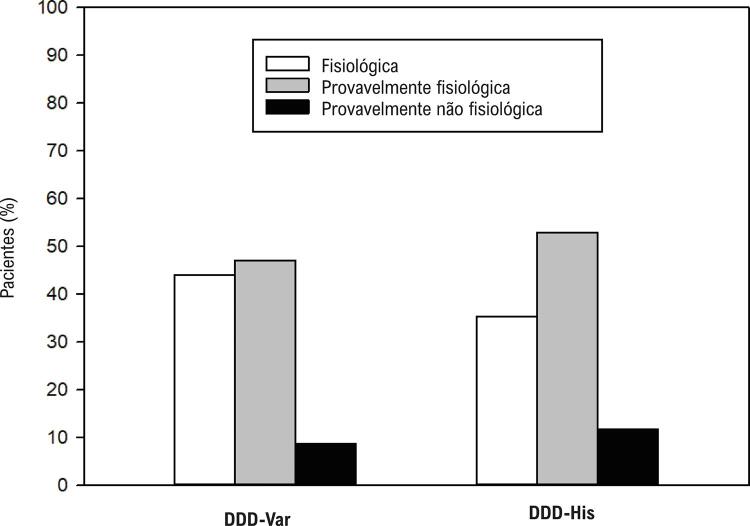
– Gráfico comparativo da sincronia cardiaca (Iimes) obtida com ambos métodos de estimulação cardíaca artificial

Como registrado na Tabela 2, ao analisarmos os critérios que sugerem captura comprovada do sistema de condução (descartando captura puramente miocárdica), 91,2% dos pacientes do grupo DDD-Var apresentaram padrão fisiológico no pós-operatório (Figura 7), enquanto 88,2% (p = 0,999) apresentaram no grupo DDD-His. O parâmetro RWPT ≥ 100 ms foi o critério que mais frequentemente descartou a possibilidade de ECA fisiológica no grupo DDD-His; no grupo DDD-Var, foi a presença de *plateau* em D1. A ECA foi catalogada como não fisiológica em três indivíduos do DDD-Var e em dois indivíduos do DDD-His.

### Duração do complexo QRS

**Figura 8:**
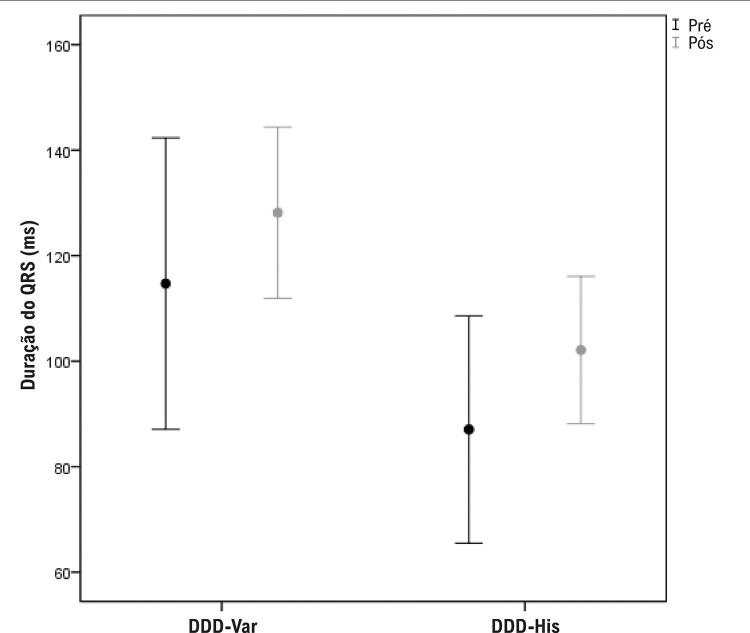
– Diferenças de duração do complexo QRS pré e pós-implante entre os grupos.

A Tabela 3 mostra que a duração média do QRS (ms) foi significativamente maior (Figura 8) no grupo DDD-Var do que no DDD-His tanto no pré (114,7 ms *versus* 87,1 ms, p = 0,001) quanto no pós-implante (128,2 ms *versus* 102,1 ms, p < 0,001). A mediana do QRS variou em 11% no grupo DDD-Var e em 20% no grupo DDD-His (p = 0,436). Quando comparada a média resultante após o implante, a duração do QRS aumentou significativamente em ambos os grupos (114,7 *versus* 128,2 ms no DDD-Var, p = 0,044; e 87,1 *versus* 102,1 ms no DDD-His, p = 0,003).

**Tabela 3:** – Comparação entre as técnicas indiretas de abordagem do feixe de His e as características pós-implante

	DDD-Var	DDD-HIS	Valor de p
	n = 34	n = 17	
QRS (ms)			
Pré-implante; média ± DP	114,7±27,6	87,1±21,6	0,001
Pós-implante; média ± DP	128,2±16,2	102,1±14,0	**< 0,001**
% variação; mediana (mínimo-máximo)	11 (-35 a 138)	20 (-14 a 66)	0,436
Tempo de Rx; mediana (mínimo-máximo)	7 (3 a 27)	21 (9 a 52)	**< 0,001**
Limiar ventricular Uni/Bi; mediana (mínimo-máximo)	0,6 (0,4 a 2,0)	0,9 (0,3 a 3,4)	0,074
Impedância ventricular Uni/Bi; média ± DP	754,8±262,2	654,9±234,1	0,190
Ondas R ventricular Uni/Bi; média ± DP	11,2±5,7	6,0±3,8	0,001
Complicações relacionadas ao implante do MPd	-	1	-

*Variáveis categóricas associadas pelo teste exato de Fisher. Variáveis quantitativas com distribuição simétrica associadas pelo teste t de Student para amostras independentes e assimétrica pelo teste de Mann-Whitney. DP: desvio padrão; MPd: marca-passo definitivo; Rx: fluoroscopia; Uni/Bipolar: estimulação unipolar ou bipolar - parâmetro mais favorável*

### Tempo de uso de Rx e parâmetros eletrônicos pós-implante

**Figura 9:**
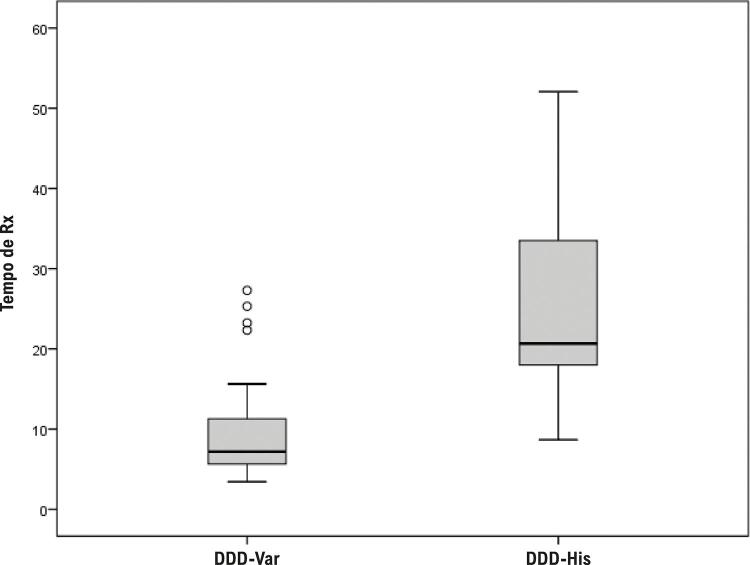
– Comparativo das médias de uso de fluoroscopia (Rx) entre os grupos.

**Figura 10:**
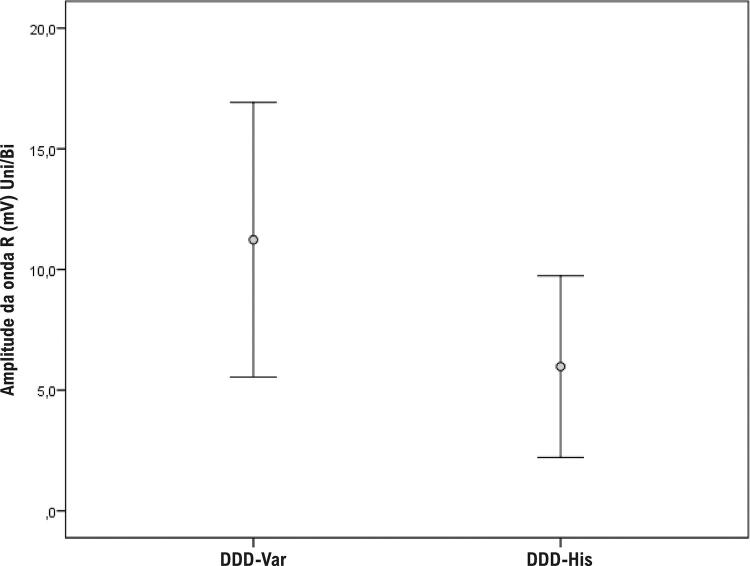
– Diferença da amplitude detectada nas ondas R entre os dois grupos.

A mediana de tempo de Rx foi significativamente menor no grupo DDD-Var (7 minutos) do que no DDD-His (21 minutos) (p < 0,001), conforme a Figura 9. Os parâmetros de estimulação apresentaram medianas e distribuições aproximadas entre os grupos (Tabela 3). O limiar para o DDD-Var foi de 0,6 V, enquanto para o DDD-His foi de 0,9 V (p = 0,074). Os valores de impedâncias ventriculares foram de 754,8 ohms para o DDD-Var e de e 654,9 ohms para o DDD-His (p = 0,19). Entretanto, a média de amplitude das ondas R (Figura 10) favoreceu significativamente o grupo DDD-Var (11,2 mV) em contraste ao DDD-His (6,0 mV) (p = 0,001).

### Acompanhamento pós-operatório (complicações agudas)

Apenas um paciente (grupo DDD-His) apresentou complicação relacionada ao MPd: pré-síncope por perda de captura por aumento de limiar do eletrodo VD.

## Discussão

A captura e ativação do feixe de His é atualmente considerada o padrão-ouro da ECA fisiológica.^8,14,24,29^ A terminologia de ECA parahissiana foi cunhada desde os primeiros resultados das tentativas de recrutar artificialmente o sistema eletrofisiológico intrínseco para reproduzir a contração cardíaca nativa.^28^ Este estudo mostra que a PHP se aproxima da captura não seletiva do feixe de His (NS-HBP) em termos de ativação homogênea ventricular (sincronia cardíaca) resultante da condução elétrica que determina a contração ventricular. Essa sincronia obtida pela técnica de PHP, confirmada por estritos critérios eletrocardiográficos e refinada pela análise da variância espacial do QRS (Synchromax®), torna a PHP uma alternativa viável, eficaz, reprodutível e de menor custo em comparação à NS-HBP.

### Sincronia cardíaca determinada pela variância espacial do QRS (IimeS)

A rápida e sincronizada transmissão do estímulo elétrico por meio da rede especializada His-Purkinje é altamente eficiente para o coração, preservando o acoplamento normal entre a condução elétrica e a contração ventricular. Zanon et al.^28^ ressaltam que a ativação ventricular obtida pela PHP pode ser aceita como resultante do recrutamento do feixe de His^30^ e demonstram que a PHP pode ativar fisiologicamente o VE, de forma semelhante à intrínseca, se conduzida por meio de um sistema His-Purkinje íntegro. Os autores avaliaram a contração do VE através da estimulação de zonas distintas do septo IV e registraram que, durante a ECA da região parahissiana (PHP), a sequência de ativação e contração resultante foi similar à nativa. A normalização (ou quase normalização) da condução intraventricular com PHP pode ser explicada através de alguns conceitos anatômicos e fisiológicos do sistema de condução e das particularidades dos seus ramos direito e esquerdo. Ao contrário do ramo direito, que não estimula o septo IV até o momento que atinge o músculo papilar anterior do VD, o ramo esquerdo tem conexões precoces com o septo IV que permitem a transmissão dos impulsos do ramo esquerdo para o septo e vice-versa. Além de explicar o porquê de a ativação septal começar no lado esquerdo, isso também explica sua expressão eletrocardiográfica (ondas Q em DI e V6 e R em V1). Quando a PHP é realizada, o estímulo artificial, de maior intensidade e maior *input* elétrico (voltagem) do que o próprio impulso fisiológico, pode passar e avançar pelas vias normais, caso não estejam danificadas. Ainda, mas menos provável, pode “pular por cima” ou transpor um eventual bloqueio e, assim, avançar, seguindo pelo sistema eletrofisiológico e pseudonormalizando a condução. A outra possibilidade, mais provável, é que o estímulo inicialmente ative o miocárdio na cúspide do septo e, na medida em que desce pela superfície septal, se espalhe normalmente pelo sistema His-Purkinje. Isso explica o alargamento inicial do QRS estimulado, expressando ativação inicial do miocárdio septal (simulando uma onda delta) e, em seguida, o rápido desenvolvimento do QRS, similar ao nativo, indicando a ativação do sistema específico e capturando o VE através das divisões da rede de Purkinje.^31^

A correlação entre ECA PHP, ativação homogênea, sincronia cardíaca e análise da variância espacial do QRS pelo IimeS também foi demonstrada por Bonomini et al.^22,32^ Segundo os autores (desenvolvedores do método Synchromax®, EXO S.A., Buenos Aires, Argentina), através de análise conjugada de variáveis como direção do impulso elétrico (da base para o ápice ou vice-versa), duração do QRS, volume e simetria ponto a ponto das curvas obtidas entre as derivações DII e V6, é possível determinar, por meio de processamento matemático instantâneo, o tempo máximo de ativação e o registro de atrasos na propagação elétrica parietal de cada câmara ventricular^22^(Figura 2A). Por meio da variância espacial do QRS, o IimeS é elaborado com valores de 0,0 a 1,0, em que 00 é sincronia perfeita e 1,00, dissincronia completa^12^(Figura 2B). A derivação DII do ECG de superfície representa a atividade do septo IV em termos do sentido e da velocidade de condução do estímulo elétrico. Da mesma forma, a derivação V6 representa a ativação da parede livre do VE. Na ausência de distúrbios de condução, a DII é positiva e apresenta duração preservada, mostrando ativação e tempo de condução fisiológicos da base do septo IV até o ápice cardíaco. Por outro lado, espera-se que a V6, ao representar o VE, seja positiva e apresente duração e volume espacial semelhantes à D2, pois segue o mesmo padrão de ativação. Graficamente, se há sincronia, observa-se sobreposição ideal das curvas resultantes (DII e V6 serão idênticas e homogêneas). A única explicação para esse efeito de curvas de ativação ventricular simultâneas e simétricas seria o recrutamento do sistema de condução intrínseco e contração ventricular coordenada e homogênea (sincronia).

Um estudo randomizado, cruzado e duplo-cego^33^mostrou que a PHP preserva a FEVE e a sincronia mecânica quando comparada à estimulação septal miocárdica do VD em pacientes com bloqueio atrioventricular de alto grau, QRS estreito e FEVE < 0,40. Kronborg et al. concluem que, nesses pacientes selecionados, não é esperado remodelamento ventricular significativo ou IC decorrentes da PHP.^33^ No mesmo contexto, outro estudo randomizado comparou 6 meses de PHP com 6 meses de estimulação apical do VD em 16 pacientes com fibrilação atrial crônica e ablação do nó AV. Os pacientes submetidos a PHP apresentaram redução da dissincronia IV, melhora na classe funcional, aumento significativo de rendimento no teste de caminhada de 6 minutos e diminuição das regurgitações mitral e tricúspide.^34^

### Técnica parahissiana PHP e NS-HBP: resultados similares de sincronia

A natureza desenhou o sistema de condução cardíaco para ativar os ventrículos do endocárdio para o epicárdio, da base para o ápice e da direita para a esquerda, considerado o “eixo fisiológico”.^13^ A presença de contração simultânea, homogênea, coordenada e simétrica demonstra sincronia. Nossos resultados confirmam que a captura comprovada do feixe de His (grupo DDD-His) resulta em contração ventricular sincrônica, idêntica à obtida durante o ritmo intrínseco, reforçando o papel da ECA do feixe de His como o padrão-ouro. A análise da variância espacial do QRS ratificou, da mesma forma, a presença de sincronia ventricular pela demonstração de IimeS < 0,4 para todos os pacientes do grupo DDD-His, exceto um (IimeS > 0,7). Nesse paciente, houve um microdeslocamento do eletrodo da posição do His, que passou a capturar o miocárdio septal adjacente – uma complicação possível dessa técnica.^10^ Quando há ativação miocárdica, similarmente ao que ocorre na ECA “convencional” do VD, tanto apical quanto puramente muscular septal, a condução elétrica se faz através do tecido inespecífico (captura miocárdica), fora da rede especializada de His-Purkinje.^2,28^ Essa modalidade indesejada de ECA apresenta um padrão ECG característico,^26^ e o resultado mecânico é uma perda da eficácia da contratilidade ventricular. Conforme exposto anteriormente, a magnitude da dissincronia é demonstrada analiticamente pelo IimeS na faixa próxima do valor 1.

O ponto primordial dos nossos resultados está na demonstração de que todos os pacientes do grupo DDD-Var que partiram de parâmetros de dissincronia (IimeS > 0,7 a 1), após o estabelecimento de PHP, recuperaram a ativação (sincronia) homogênea dos ventrículos (92,1% com IimeS < 0,4; o restante, IimeS = 0,4 a 0,69. Variação mediana do IimeS no grupo DDD-Var = -74). Todavia, reforçando ainda mais as semelhanças entre a ECA PHP e a NS-HBP, o sentido da ativação elétrica ventricular produzida na totalidade dos casos para ambos os grupos se enquadrou nas categorias de eixo fisiológico, na verificação integral dos critérios de Mala et al.^25^ e eixo provavelmente fisiológico, na falta de um dos critérios.

### Duração dos QRS estimulados

O funcionamento adequado da “bomba cardíaca” depende de um sistema eletromecânico bastante coordenado (síncrono). Anormalidades de condução, como a provocada pela estimulação puramente miocárdica ventricular, causam dissincronia, que pode ter consequências deletérias (miocardiopatia associada à ECA).^3,4,35^ A PHP utilizada neste estudo, apesar de ter corrigido a dissincronia em praticamente todos os pacientes, o fez à custa de um alargamento significativo do QRS (Figura 8). Mesmo assim, nenhum caso superou o valor crítico de 150 ms.^19,28^ Um estudo conduzido por Zhang et al.^9^ que objetivou avaliar o efeito agudo da ECA seletiva do feixe de His (S-HBP), da NS-HBP e da estimulação muscular do septo IV direito na sincronia elétrica e mecânica do VE, mostrou que tanto a S-HBP quanto a NS-HBP poderiam restaurar a contração elétrica fisiológica e a sincronia mecânica ventricular. Ratifica-se que a estimulação do feixe de His, em qualquer das suas modalidades, pode manter a ativação ventricular nativa através do sistema de condução intrínseca, o que é comprovadamente mais fisiológico e caracterizado por melhores indicadores de sincronia cardíaca do que pacientes com ECA puramente muscular.^9^ Contudo, em vários estudos, a duração do QRS no ECG de 12 derivações tem sido usada como um marcador indireto de sincronia elétrica, e a prolongação do QRS, intrínseco ou estimulado, foi associada a risco aumentado de insuficiência cardíaca.^8,36^ Em nosso estudo, houve diferença a favor do DDD-His quando comparado ao DDD-Var (PHP) em relação à menor duração do QRS estimulado; porém, em ambas as técnicas, percebeu-se alargamento significativo e na mesma magnitude quando comparado ao QRS intrínseco. Esse resultado também esteve presente no estudo de Zhang et al.^9^ durante a ECA de baixa voltagem com NS-HBP, em que, apesar de ter demonstrado melhor sincronia elétrica e mecânica do que a estimulação septal muscular, o QRS estimulado era mais largo do que o QRS intrínseco.

Todavia, nossos achados destacam que a magnitude da variação do QRS pré e pós-procedimento entre os grupos não foi significativa (p = 0,436), ressaltando que a NS-HBP (grupo DDD-His) comprovadamente capturava o sistema elétrico nativo. A explicação seria que o estímulo próximo ao feixe de His e não diretamente sobre ele (ECA fisiológica indireta) produz complexos QRS fusionados. Observou-se alargamento inicial (onda pseudodelta) atribuível à captura concomitante do tecido muscular perihissiano, resultando em duas frentes despolarizantes que se fusionam. Uma frente recruta o sistema intrínseco e ativa o VE pelo ramo esquerdo nativo, e a outra trafega brevemente pelo miocárdio do septo IV adjacente ao His até encontrar e ativar o sistema de condução pelo lado direito, similarmente ao observado nas síndromes de pré-excitação com vias acessórias parahissianas.^31^ Contudo, embora a presença de dissincronia mecânica seja frequente em pacientes com complexos QRS largos, a largura do QRS por si só não parece ser um marcador eficiente de diagnóstico de dissincronia.^12,13,22^ O eixo de ativação e a dispersão morfológica da despolarização ventricular seriam características mais marcantes, como mostrado por Bonomini et al.^22^ Essa importante mudança de paradigma foi confirmada em uma publicação comparando o método de variância espacial do QRS com a ecocardiografia. O ECG com análise de variância teria sensibilidade superior e relevante valor preditivo negativo para a detecção de dissincronia mecânica quando comparado somente à duração do QRS do ECG convencional^32^ sugerindo que importa mais a “coordenação” do que a duração do QRS.

### Estimulação cardíaca fisiológica com ambas as técnicas?

Publicações recentes^26,27,37^ permitem discernir, no ECG, as diferenças entre ativação puramente miocárdica e a captura direta ou indireta do sistema de condução intrínseco (Figura 3). A análise desses critérios, como neste estudo, reduziria o risco de incorretamente identificar a captura do miocárdio inespecífico como PHP, o que, em alguns casos, está associada a resultados clínicos semelhantes à ECA convencional do VD.^26,28^ Quando a condução elétrica trafega pelo tecido muscular não especializado, ativa o septo IV de forma anômala e leva ao atraso acentuado da ativação da parede lateral do VE, ocasionando alteração morfométrica e deformação do QRS. Pela aplicação exaustiva desses princípios eletrocardiográficos,^26,27^ não houve diferença significativa (p = 0,999) entre os grupos estudados. De interesse, no grupo DDD-Var, a ausência de entalhe na derivação V1 e o tempo entre o estímulo (espícula) até o RWPT em V6 < 100 ms foram as duas características mais marcantes ao conceito de ECA fisiológica, assemelhando a ECA PHP à NS-HBP. Em contrapartida, no DDD-His, talvez corroborando a manifestação de NS-HBP, o achado que mais afastou o diagnóstico de ECA fisiológica foi RWPT > 100 ms. Contudo, isso é explicado pelo tempo de propagação do estímulo até a penetração e captura do feixe de His. O método de análise da variância espacial do QRS (Synchromax®) corrobora de forma coerente essa sincronia, comparável ao NS-HBP e determinando um IimeS < 0,4 com sobreposição das curvas de ativação.

### Segurança e eficácia da PHP

Devido às características da curva de aprendizado relacionada à técnica da ECA do feixe de His, preferiu-se aplicar essa estratégia em pacientes com DNS e condução atrioventricular preservada. Os pacientes com bloqueios intra e infra-His apresentam desafios adicionais à essa técnica e, muitas vezes, precisam que a estimulação do VD seja garantida por um segundo eletrodo de *backup* (maior consumo de recursos e maior risco de complicações).

O posicionamento do eletrodo VD nas regiões proximais do septo IV na busca de ativação parahissiana é mais simples e de fácil reprodutibilidade, exequível em qualquer serviço que realize implantes de MPd com o auxílio da anatomia radiológica.^19^ Nossos resultados confirmam que a PHP é viável e especialmente segura para pacientes dependentes da ECA, grupo no qual a estimulação por captura do His pode ser mais desafiadora.^10^ Ainda, a PHP possui o benefício de apresentar uma curva de aprendizado mais breve e um tempo de exposição à Rx durante o procedimento significativamente menor (Figura 9).

Observa-se, também, que a programação do MPd e a solução de problemas intraoperatórios relacionados à ECA direta do His (HBP) podem ser um obstáculo.^37^ Em contrapartida, para o grupo de pacientes DDD-Var, foram registrados parâmetros eletrônicos favoráveis, como ondas R de amplitude significativamente melhor (Figura 10). É possível vislumbrar que a PHP, realizada com eficácia e segurança, supera algumas clássicas inconveniências da ECA fisiológica quando comparada à HBP. Hanifin et al.^37^ sugerem que os operadores que praticam HBP necessitam de um treinamento específico para a resolução de adversidades tanto durante o procedimento quanto durante os ajustes de programação do dispositivo. Naturalmente, esse cenário aumenta o consumo de recursos de saúde.^37^

Durante a HBP, a baixa amplitude da onda R pode ser uma realidade indesejada e, dessa forma, predispor a problemas de detecção da atividade elétrica intrínseca, acarretando disfunção do MPd e conflitos de programação.^37^ Observa-se, também, que a captura direta do feixe de His habitualmente requer maior energia de saída (voltagem) e, consequentemente, resulta em drenagem precoce da bateria do gerador.^10^ Em nosso estudo, foi feita procura exaustiva por parâmetros adequados de ECA, o que pode ter interferido no aumento do tempo de Rx nesse grupo.

### Revisão da classificação da estimulação cardíaca fisiológica direta e indireta

Nosso estudo é pioneiro em comparar a sincronia eletromecânica do VE através do processamento instantâneo da variância espacial do QRS entre pacientes submetidos a NS-HBP e aqueles submetidos a ECA PHP. Considerando que a ausência de diferença não significa estritamente a equivalência, propomos, a partir dos nossos achados, reclassificar a ECA fisiológica com base no grau de envolvimento (captura) direto ou indireto do sistema His-Purkinje. Dessa forma, a ECA fisiológica direta integraria a captura rigorosa sob mapeamento do sistema elétrico intrínseco, com demonstração do recrutamento do feixe de His (S-HBP – His direto seletivo) ou um de seus ramos (estimulação do ramo esquerdo – técnica *deep-septal*). A ECA fisiológica indireta seria representada pela NS-HBP e pela PHP, sob comprovação de ativação ventricular rápida e homogênea (sincrônica), apresentando, no entanto, captura breve, parcial e variável do miocárdio perihissiano (configurando onda pseudodelta no ECG). Apropriadamente, a ECA fisiológica indireta contemplaria a variante anatômica intrasseptal do His (tipo II),^24,37,40,41^ que produz NS-HBP em quase todos os casos e pode estar presente em mais de 30% das apresentações anatômicas.

Por fim, a estimulação direta do feixe de His é o padrão-ouro para preservar o padrão de ativação fisiológica. As formas indiretas não seletivas e a PHP são variantes que, analogamente, como comprovado neste estudo, preservam a sincronia ventricular contrátil, evitando os potenciais efeitos deletérios da ECA convencional.^9,12^

Conforme apresentado, a ECA fisiológica indireta do tipo PHP é capaz de recrutar precocemente o sistema de condução intrínseco.^31^ Estamos diante de uma modalidade de ECA interessante e promissora, e o uso de ferramentas como a análise de sincronia pela variância espacial do QRS (IimeS, Synchromax®) torna o método mais facilmente reprodutível e eficaz.

### Limitações

Este estudo contemplou uma série relativamente pequena de pacientes com indicações heterogêneas de ECA, muito embora os testes para a análise de sincronia tenham sido realizados com sobre-estimulação e captura ventricular uniforme (modo VVI). Além disso, é um estudo unicêntrico com limitações na análise retrospectiva dos dados. Ainda, deve-se considerar que, apesar das específicas condições metodológicas, a conferência de sincronia foi por método indireto, não tendo sido consideradas outras variáveis que possam alterar a condução elétrica cardíaca. Por fim, o efeito real da manutenção da sincronia, evitando a miocardiopatia pela ECA, só poderia ser avaliado através de seguimento de longo prazo, o que não era o objetivo deste estudo. Seu foco e fortaleza estão na comparação de estratégias de ECA fisiológica durante o processo perioperatório de implante.

## Conclusão

Este trabalhoevidencia a manutenção da sincronia cardíaca com a PHP similar à NS-HBP, agrupando-as em uma nova classificação: ECA fisiológica indireta. Embora essa estratégia seja promissora e atraente, apresentando-se como uma alternativa válida e comparável quando realizada com rigor metodológico, tanto o emprego da análise eletrocardiográfica da variância espacial do QRS quanto a comprovação indireta de captura do sistema de condução, como neste estudo, precisam ser validados, como qualquer nova tecnologia ou procedimento, com novos estudos e um número maior de pacientes.
