# Supplementary material for: Sincronia Ventricular na Estimulação Cardíaca Parahissiana: Alternativa por Ativação Cardíaca Fisiológica (Estimulação Indireta do Feixe de His)?
Source: Arq Bras Cardiol. 2021 Dec 20;118(2):488–502. [Article in Portuguese] doi: 10.36660/abc.20201233 (PMC8856677; doi:10.36660/abc.20201233)
Supplement: Translation

# Translation: Ventricular Synchrony in Para-Hisian Cardiac Pacing as an Alternative for Physiological Cardiac Activation (Indirect Recruitment of the His Bundle?)

**Authors:** Andres Di Leoni Ferrari, Guilherme Ferreira Gazzoni, Luis Manuel Ley Domingues, Jessica Caroline Feltrin Willes, Gustavo Chiari Cabral, Flavio Vinicius Costa Ferreira, Laura Orlandini Lodi, Gustavo Reis

**Affiliations:** 1 Brazil Serviço de Cardiologia. Hospital São Lucas da Pontifícia Universidade Católica do Rio Grande do Sul (PUCRS), Porto Alegre, RS – Brazil; 2 Mexico Universidad Popular Autonoma del Estado de Puebla - Facultad de Medicina, Puebla – Mexico; 3 Brazil Eletrofisiologia Londrina, Londrina, PR – Brazil

**Keywords:** Artificial Pacemaker, Artificial Cardiac Pacing, Electric Stimulation Therapy

## Abstract

**Background:**

Artificial cardiac pacing by direct or indirect His bundle capture results in synchronous ventricular contraction (physiological pacing).

**Objectives:**

To compare cardiac synchronization, technical characteristics, and electronic parameters between two techniques of indirect His-bundle pacing: non-selective (NS-HBP) vs para-Hisian pacing (PHP).

**Methods:**

The experimental intervention (between November 2019 and April 2020) consisted of implanting a DDD pacemaker in patients who had left ventricular ejection fraction (LVEF) > 35%. The resulting cardiac synchronization was compared using an electrocardiographic algorithm that analyzed QRS variation and the technical characteristics of non-selective Hisian pacing (DDD-His) and para-Hisian pacing (DDD-Var).

**Results:**

Of 51 total patients (men: 28), 66.7% (34) were allocated to the DDD-Var group and 33.3% (17) to the DDD-His group. The mean ages in each group were 74 and 79 years, respectively. In the DDD-Var group, QRS variation (ventricular synchrony) improved after implantation (p < 0.001). In post-implantation ECG, 91.2% of the DDD-Var group presented a physiological pacing pattern, which was similar to the DDD-His group (88.2%; p = 0.999). The paced QRS axis was also similar (physiological) for both groups. Intraoperative fluoroscopy time (XRay) during implantation was lower for the para-Hisian technique (median 7 min in the DDD-Var group vs 21 min in the DDD-His group, p < 0.001). The mean QRS duration increased in the DDD-Var group (114.7 ms pre-implantation vs 128.2 ms post-implantation, p = 0.044). The mean post-implantation R-wave amplitude was 11.2 mV in the DDD-Var group vs 6.0 mV in the DDD-His group, p = 0.001.

**Conclusion:**

Para-Hisian pacing appears to indirectly recruit the His bundle, which would make this an effective and comparable strategy for physiological pacing, resulting in synchronous ventricular contraction similar to that of non-selective Hisian pacing.

## Introduction

The evolution of artificial cardiac pacing has shown that impulse conduction through non-physiological muscle activation of the right ventricle (RV), especially apical pacing (“conventional” pacing), is associated with deleterious cardiac effects and negative clinical repercussions.^1^ Although conventional pacing resolves the electrical and hemodynamic problem by restoring heart rate, it comes at the expense of electromechanical changes resulting from “cardiac dyssynchrony”.^5^ Dyssynchrony manifests electrically (a wide QRS with left bundle branch block pattern) and mechanically (cardiac remodeling, mitral regurgitation and systolic dysfunction).^4,5^

Several studies have confirmed the feasibility and positive clinical results of direct His-bundle pacing compared to conventional pacing.^6^ Currently, direct His-bundle pacing can be considered for almost all cardiac conduction disorders. Standardizing this technique, however, is challenging. Some criteria must still be refined, such as the clinical differences, if any, between selective (S-HBP) and non-selective His-bundle pacing (NS-HBP),^9^ higher capture thresholds, which result in accelerated generator battery depletion; and the additional resources (specific leads and sheaths) required for positioning the ventricular lead in contact with the His bundle.^10,11^ There is also quite long learning curve, with procedures of increased duration, success rates between 60% and 90% and, in some cases, programming difficulties.^10,12^ Para-Hisian pacing (PHP), which has a shorter learning curve and a lower cost in terms of materials, can also preserve the synchrony of ventricular depolarization.^12,13^ The technique consists of placing the ventricular lead in the uppermost proximal region of the right side of the interventricular (IV) septum, adjacent to the conduction system. Being more reproducible, this technique is a promising alternative to physiological cardiac pacing by indirectly and rapidly recruiting the His-Purkinje system, similar to NS-HBP.^12^ The aim of this study was to perform a comparative analysis of the cardiac synchronization obtained through the NS-HBP and PHP techniques, indirectly capture the conduction system for physiological pacing.

## Methodology

This experimental intervention study was conducted at the Cardiac Pacing Unit and Pacemaker Outpatient Clinic, Hospital São Lucas, Pontifical Catholic University of Rio Grande do Sul, Porto Alegre, Brazil. The sample was selected from patients undergoing implantation of a permanent dual-chamber pacemaker (DDD pacing mode) according to current guidelines^14^ who had mid-range (36-49%) or preserved left ventricular ejection fraction (LVEF) (>50%).^15^ All implant procedures were performed by the same main operator (ADLF). All participants provided written informed consent prior to inclusion. Patients indicated for implantation of a cardiac defibrillator, cardiac resynchronization therapy (CRT) candidates, a single-chamber pacemaker, and those with incomplete data were excluded.

The patients were divided into two groups: DDD-Var (RV lead implantation for PHP) and DDD-His (RV electrode positioned for NS-HBP), guided by conventional electrophysiological mapping.

The technique for positioning the RV lead in a uppermost position to the IV septum for PHP followed previously described methodology.^5,16^ Briefly summarized, the ventricular lead (conventional bipolar cables with active fixation – from any manufacturer) was mounted to a manually customized stylet with a wide curvature in the distal third followed by a more accentuated posterior curvature in the proximal portion (Figure 1).^5,12^

**Figure 1:** – Left: a hand-shaped stylet guiding the positioning of the RV lead in the uupermost proximal third of the interventricular septum for para-Hisian pacing. Center: Operator (ADLF) showing a comparison of the shape obtained by molding the guide wire with the curvature of one of the pre-molded sheaths available in Brazil (C315His Medtronic™). Right: fluoroscopy (left oblique projection) showing the final position of the lead in the right ventricle. Note the angulation of the tip, which is perpendicular to the spine. Adapted from^12,19^

Guided by radiological anatomy (posteroanterior view), the lead was advanced to the pulmonary artery and, with the guide wire fully inserted, it was pulled into the RV outflow tract. In this view, the interventricular septum is divided into 3 zones:^19^ the cranial third of the RV (between the prominence of the pulmonary artery and the roof of the tricuspid valve), the medial third, and the lower lower third. Septal positioning was then confirmed by radioscopy of the left anterior oblique view (30 to 45 degrees). In this view, the the lead is oriented pointing perpendicularly to the spine, in a direction opposite the RV free wall^12,19,20^(Figure 1).

To confirm that PHP capture had occurred in the DDD-Var group, the narrowest QRS complex was sought (≤ 130 ms; always < 150 ms) by mapping the IV septum with a ventricular lead^21^ prior to releasing the screw-in. Simultaneously, in real (intraoperative) time, under VVI pacing decreasing from an amplitude of 5 V and a pulse width of 1 ms, QRS variation analysis with the Synchromax® system (EXO, Buenos Aires, Argentina) determined the immediate Synchrony Index (imeSI). The PHP site with the best index was chosen for definitive fixation of the RV lead.

The imeSI is a result of graphic and mathematical processing of the signal averaged by the cross-variation of the DII (right interventricular septum) and V6 (lateral wall of the left ventricle [LV]) leads. For this analysis, Synchromax® uses the measurement of the flow of electric current (volume and direction) and the agreement analysis of the intrinsicoid deflection of the QRS (Figure 2A).^12,22,23^ ImeSI values < 0.40, > 0.41, and < 0.69, > 0.7 indicate synchrony, moderate dyssynchrony, and severe dyssynchrony, respectively (Figure 2B).^11,19,21^

**Figure 2A:** – Correlation of QRS variation and normal values for a patient with intact intraventricular conduction (leads II and V6). Left: Conventional ECG traces. Center: overlapping QRS segments (D2 QRS and V6 QRS). Right: Cross-correlation analysis of leads II and V6. The QRS peaks coincide and the maximum cross-correlation signal is at time zero (CorS = 0). CorS: cross-correlation offset (ms). CorW: cross-correlation width (ms), CorA: cross-correlation amplitude (mV), AII: area under lead D2, aV6: area under lead V6. Adapted from Bonomini et al.^22^

**Figure 2B:** – Curves obtained with Synchromax^TM^ according to the immediate synchrony index performed in real time from the pacing site in relation to cardiac synchrony obtained from the RV pacing site. Blue lines: QRS variation analysis from lead II. Red dashes: QRS variation analysis for lead V_6_.CRT: cardiac resynchronization therapy; LAH: left anterior hemiblock; RV: right ventricle. LBBB: Left bundle branch block. RBB: Right bundle block

For His-bundle capture (DDD-His group): a) a quadripolar catheter is introduced via the femoral artery to perform electrophysiological mapping and record His-bundle potentials; b) a dedicated sheath (C315-His, Medtronic, Minneapolis, MN, USA) is introduced via the cephalic or subclavian vein to position the lumenless SelectSecure MRI SureScan Model 3830 lead (Medtronic) into the His topography, which is indicated by the electrophysiology catheter. Selective (S-HBP) or non-selective (NS-HBP) His-bundle capture was then confirmed.^24^ Intraoperatively, while VVI pacing decreased from a 5 V pulse amplitude and a 1 ms pulse width, noninvasive QRS spatial variance analysis (Synchromax®, EXO, Buenos Aires, Argentina), determined the imeSI in real time through the same methodology described above (PHP pacing). The best NS-HBP values were selected for analysis.

In addition to the imeSI, intraoperative fluoroscopy time (Xray) and surface electrocardiogram (ECG) during the procedure and prior to discharge were recorded for both groups. Local endocardial activation of the RV was confirmed through R wave amplitude measurement, while the unipolar and bipolar capture threshold and impedance were determined through a decremental pacing test in VVI mode. The best values were selected for analysis.

ECG analysis was blinded to operators during the course of the methodology and to the pre- and postoperative characteristics of the implantation and the patient. To determine the electrical axis of the QRS complex in the postoperative ECG, physiological pacing considered the presence of ventricular activation from right to left (QRS [+] in leads D1 and aVL) and from top to bottom (QRS [+] in DII, DIII, and aVF), as well as a transition (R wave > S wave) to V3-V4 in the precordial leads.^25^ The presence of all three criteria was considered a “physiological” axis, the presence of two criteria was considered “probably physiological”, while the presence of only one or none was considered “non-physiological”.

In the DDD-Var cases, to confirm that synchronous PHP was correlated with NS-HBP (which ruled out pure myocardial ventricular pacing), we used the electrocardiographic model of Burri et al.,^26^ (Figure 3) verifying an absence of plateau and notching in leads D1 and V1, respectively, as well as an R wave peak time (RWPT) < 100 ms in V6.^26^ The presence of these three parameters indicated “physiological” pacing similar to NS-HBP and rules out purely ventricular activation. The pacing can be considered “probably physiological” when 2 of these criteria are present, “probably non-physiological” pacing when 1 is present, and merely myocardial pacing when none are present.

**Figure 3:** – Electrocardiographic model proposed by Burri et al.^26^ including a combination of: a) absence of plateau in D1; b) absence of notching in lead V1; c) R-wave peak time in V6 < 100 ms.^28–30^ The presence of a, b, and c indicates “physiological” pacing in NS-HBP and rule out purely myocardial activation. The presence of 2 of criteria indicates “probably physiological” pacing, while the presence of only one criterion indicates “probably not physiological” pacing. The absence of all criteria indicates purely nonspecific myocardial capture (myocardial pacing).^28^

For qualitative analysis, the acute clinical course (until hospital discharge) of all patients was followed regarding cardiovascular complications, especially those related to pacemaker implantation.

### Statistical analysis

Categorical variables were analyzed using Fisher’s exact test or the chi-square test with Yates correction, depending on the frequency distribution in different categories, and were described as frequencies and percentages. The McNemar test was used for pre- and postoperative comparisons of categorical variables. Symmetrically distributed quantitative variables were compared between groups using Student’s *t*-test for independent samples and within groups using Student’s *t*-test for paired samples. Asymmetrically distributed variables were compared within groups using the Mann-Whitney test and the Wilcoxon test. The Kolmogorov-Smirnov test was used to analyze quantitative variables, which were described as mean and standard deviation if symmetrically distributed or by the median, minimum, and maximum value if asymmetrically distributed. A 5% significance level was used for the comparisons. Microsoft Excel was used to compile the data, which was subsequently analyzed in SPSS v. 20.0.

## Results

Between November 2019 and April 2020, 51 patients, the majority (28) being men, were included in the sample: 34 in the DDD-Var group and 17 in the DDD-His group, whose mean ages were 74 and 79 years, respectively. The most prevalent etiology for pacemaker implantation was complete atrioventricular block in the DDD-Var group and sinus node dysfunction in the DDD-His group. LVEF was preserved (> 50%) in 40 patients and intermediate (36%-49%) in 11 patients. The groups are compared in Table 1.

**Table 1:** – Comparison of group characteristics

	DDD-Var n=34	DDD-His n=17	p
Male, n(%)	21 (61.8)	7 (41.2)	0.274
Age in years, mean ± SD	74.0±8.9	79.0±7.9	0.063
**Underlying disease, n(%)**			**0.004**
Complete AVB	17 (50.0) ^a^	3 (17.6) ^b^	
Second-degree AVB	9 (26.5) ^a^	3 (17.6) ^a^	
Sinus node dysfunction	8 (23.5) ^a^	11 (64.7) ^b^	
Preserved ejection fraction (>50%), n(%)	27 (90.0)	13 (86.7)	0.999

*SD: standard deviation. Associations between categorical variables were tested with Fisher's exact test or the chi-square test with Yates correction, while associations between quantitative variables were tested with Student's t-test for independent samples. a, b: different letters indicate significantly different percentages.*

### Cardiac synchronization

QRS analysis (Synchromax®) revealed a significant difference (p<0.001) in imeSI pre- and postoperatively. Of the 20 patients who were synchronous in the preoperative period, 19 (95.0%) remained synchronous in the postoperative period. Most of the remaining 31 patients were dyssynchronous (26, imeSI >0.7; 5 imeSI 0.41-0.69). Of these, 30 (96.8%) became synchronous after implantation, with only 1 maintaining an intermediate imeSI.

There was also a significant variation in imeSI (p<0.001) between pre- and post-implantation the DDD-Var group. Of 26 dyssynchronous patients, 25 (96.2%) became synchronous and only 1 (3.8%) remained intermediate. According to the imeSI, all 8 synchronous patients in the preoperative period remained synchronous after implantation. In the DDD-His group, 11 of the 12 individuals (91.7%) who were synchronous remained synchronous, with 1 was classified as dyssynchronous in the postoperative period. All 5 remaining patients (dyssynchronous or moderately dyssynchronous) became synchronous after implantation (Table 2).

**Table 2:** – Comparison of results before and after permanent pacemaker implantation

	DDD-Var	DDD-His	p
**Synchrony index**	n=34	n=17	
Preintervention^*^; n(%)			0.001
Synchronous	8 (23.5)^a^	12 (70.6)^b^	
Intermediate	3 (8.8)^a^	2 (11.8)^a^	
Asynchronous	23 (67.6)^a^	3 (17.6)^b^	
**Postintervention; n(%)**			0.560
Synchronous	33 (97.1)	16 (94.1)	
Intermediate	1 (2.9)	-	
Asynchronous	-	1 (5.9)	
**imeSI value**			
Pre^*^; median (min-max)	1.00 (0.12 to 1.00)	0.21 (0.06 to 1.00)	**0.001**
Post; median (min-max)	0.18 (0.11 to 0.70)	0.18 (0.11 to 0.72)	0.461
%variation^**^; median (min-max)	-74 (-89 to 192)	0 (-77 to 243)	**<0.001**
			
Post-implantation ECG	n=34	n=17	
Axis; n(%)			0.074
Physiological	26 (76.5)	8 (47.1)	
Probably physiological	8 (23.5)	9 (52.9)	
Pacing	n=34	n=17	
Category (%)			0.915
Physiological	15 (44.1)	6 (35.3)	
Probably physiological	16 (47.1)	9 (52.9)	
Probably not physiological	3 (8.8)	2 (11.8)	
			
**Missing physiological pacing criterion; n(%)**	n=19	n=11	
RWPT ≥100 ms	5 (26.3)	6 (54.5)	0.238
Plateau in lead D1	12 (63.2)	4 (36.4)	0.299
Notching in lead V1	5 (27.8)	3 (27.3)	0.999

AVB: atrioventricular block; ECG; electrocardiogram; imeSI: immediate synchrony index; RWPT: R-wave peak time. Synchronous: imeSI ≤ 0.40; intermediate: imeSI 0.41-0.70; asynchronous: imeSI ≥ 0.71. Associations between categorical variables were tested with Fisher's exact test or the chi-square test with Yates correction, while associations between quantitative variables with asymmetric distribution were tested with the Mann Whitney test. **% variation=([post value - pre value]/pre value*100); a,b: different letters indicate significantly different percentages.

Table 2 also describes significant differences between groups in the preoperative period: the DDD-Var group had more dyssynchronous patients (67.6% vs. 17.6% in the DDD-His group) and fewer synchronous patients (23.5% vs. 70.6% in the DDD-His group). Postoperatively, the groups were similar, since overall synchrony was achieved in both groups (97.1% in the DDD-Var group vs 94.1% in the DDD-His group; p = 0.560)(Figure 4). The imeSI differed significantly between the groups preoperatively (1.00 vs 0.21, p=0.001) but not postoperatively (0.18 vs 0.18, p = 0.461)(Figure 5), confirming that both PHP and NS-HBP achieved physiological pacing. The median imeSI reduction in the DDD-Var group was 74% (vs a median of 0% in the DDD-His group, p<0.001), indicating the magnitude of the correction. Analyzing each group separately and comparing the synchrony data between the pre- and postoperative periods, the DDD-Var group varied significantly (median 1.00 vs. 0.18 in the pre- and postoperative periods, respectively; p < 0.001) and, as expected, there was no significant difference in the DDD-His group (median 0.21 vs 0.18 in the pre- and postoperative periods, respectively; p = 0.453).

**Figure 4:** – Distribution frequency of pre- and postoperative cardiac synchronization categories between the para-Hisian pacing (DDD-Var) and non-selective His pacing (DDD-His) groups.

**Figure 5:** – Comparison of the immediate synchrony index between the para-Hisian pacing (DDD-Var) and non-selective His pacing (DDD-His) groups.

### Physiological axis

Figure 6 shows the similar post-implantation QRS electrical axes in both groups (p=0.074). Corroborating the methods’ similarity in His-Purkinje conduction system recruitment, there was no difference (p=0.915) between the “probably physiological” (47.1% DDD-Var vs. 52.9% DDD-His) and “physiological” (44.1% vs. 35.3%, respectively) results.

**Figure 6:** – Comparison of the ECG axis between the para-Hisian pacing (DDD-Var) and non-selective His pacing (DDD-His) groups.

### Physiological Pacing - Criteria for Conduction System Capture

As shown in Table 2, regarding the criteria for conduction system capture (excluding purely myocardial capture), 91.2% and 88.2% of the DDD-Var and DDD-His groups had a physiological pattern in the postoperative period (p = 0.999) (Figure 7). The criteria that most frequently confounded physiological pacing were an R wave peak time (RWPT) ≥ 100 ms in the DDD-His group and a plateau in D1 in the DDD-Var group. Pacing was classified as “non-physiological” in 3 DDD-Var patients and 2 DDD-His patients.

**Figure 7:** – Comparative chart of cardiac synchrony (Iimes) obtained with both methods of artificial cardiac stimulation.

### QRS complex duration

Table 3 shows that the mean QRS duration (ms) was significantly higher (Figure 8) in the DDD-Var group than the DDD-His group, in both the pre-implantation (114.7 vs 87.1 ms, p = 0.001) and post-implantation periods (128.2 vs 102.1 ms, p < 0.001). The QRS varied by a median of 11% in the DDD-Var group and 20% in the DDD-His group (p=0.436). Compared to the post-implantation mean, QRS duration significantly increased in both groups (DDD-Var: 114.7 vs 128.2 ms, p = 0.044; DDD-His group: 87.1 vs 102.1 ms, p = 0.003).

**Table 3:** – Comparison of indirect techniques of His bundle capture and post-implantation characteristics

	DDD-Var	DDD-His	p
	n=34	n=17	
QRS ms			
Preintervention; mean±SD	114.7±27.6	87.1±21.6	0.001
Postintervention; mean±SD	128.2±16.2	102.1±14.0	<0.001
%variation; median (min-max)	11 (-35 to 138)	20 (-14 to 66)	0.436
Fluoroscopy time; median (min-max)	7 (3-27)	21 (9-52)	<0.001
Uni/Bi ventricular threshold; median (min-max)	0.6 (0.4-2.0)	0.9 (0.3-3.4)	0.074
Uni/Bi ventricular Impedance; mean ± SD	754.8±262.2	654.9±234.1	0.190
Uni/Bi ventricular R waves; mean ± SD	11.2±5.7	6.0±3.8	0.001
Complications related to pacemaker implantation	-	1	-

*Associations between categorical variables were tested with Fisher's exact test; associations between quantitative variables with symmetric distribution were tested with Student's t-test for independent samples; variables with asymmetric distribution were tested with the Mann-Whitney test.*

**Figure 8:** – Pre- and post-implantation differences in QRS complex duration between the para-Hisian pacing (DDD-Var) and non-selective His pacing (DDD-His) groups.

### Fluoroscopy time and post-implantation electronic parameters

As shown in Figure 9, the median fluoroscopy time was significantly shorter in the DDD-Var group (7 vs 21 min, p < 0.001). The medians and distributions of pacing parameters were similar between groups (Table 3): the mean ventricular threshold was 0.6 V vs 0.9 V in the DDD-Var and DDD-His groups, respectively (p=0.074), while the mean ventricular impedance was 754.8 ohms vs 654.9 ohms in the DDD-Var and DDD-His groups, respectively (p=0.19). However, the mean R wave amplitude (Figure 10) was significantly better in the DDD-Var group than the DDD-His group (11.2 mV vs 6.0 mV, p = 0.001).

**Figure 9:** – Comparison of mean intraoperative XRay time between the para-Hisian pacing (DDD-Var) and non-selective His pacing (DDD-His) groups.

**Figure 10:** – Difference in R wave amplitude between the para-Hisian pacing (DDD-Var) and non-selective His pacing (DDD-His) groups.

### Postoperative follow-up (acute complications)

Only one patient (DDD-His group) had a pacemaker-related complication (near-syncope due to acute RV pacing threshold).

## Discussion

His-bundle capture and activation is now the gold standard for physiological pacing.^8,14,24,29^ The term PHP was coined after the first attempts to artificially recruit intrinsic electrophysiological activity to reproduce native cardiac contraction.^28^ This study shows that PHP is non-inferior and quite similarly approximates to NS-HBP in terms of homogeneous ventricular activation (cardiac synchronization), which results from the electrical conduction that determines ventricular contraction. The synchronization obtained through the PHP technique, confirmed by strict ECG criteria and refined by QRS spatial analysis (Synchromax®), makes PHP a viable, effective, reproducible, and lower cost alternative to NS-HBP.

### Cardiac synchronization determined by Spatial variance analysis of the QRS (imeSI)

The rapid and synchronized transmission of electrical stimuli through the specialized His-Purkinje network is highly efficient for the heart, preserving the normal coupling between electrical conduction and ventricular contraction. Zanon et al.^28^ point out that the ventricular activation obtained by PHP is acceptable since it results from recruitment of the His bundle,^30^ demonstrating that PHP can physiologically activate the LV like natural conduction does through a healthy His-Purkinje system. These authors evaluated LV contraction by pacing distinct zones of the interventricular septum, reporting that during pacing of the para-Hisian region (PHP), the resulting activation and contraction sequence was similar to the natural sequence. The normalization (or near normalization) of intraventricular conduction with PHP can be explained due to certain concepts related to the anatomy and physiology of the conduction system and the particularities of its right and left branches. Unlike the right branch, which does not stimulate the IV septum until it reaches the RV anterior papillary muscle, the connections between the left branch and the IV septum allow the transmission of impulses from the left branch to the septum and vice versa. This explains why both septal activation and its ECG expression begin on the left side (Q waves in D1 and V6 and R waves in V1). When PHP pacing is performed, it could be that the artificial stimulus, which is of greater intensity and greater electrical input (voltage) than the physiological impulse, advances through the normal pathways if they are not damaged or, less likely, “skips” or overcomes blockages, continuing forward through the electrophysiological system, achieving pseudonormalized conduction. The other, more likely, possibility is that the stimulus initially activates the myocardium in the septal cusp and, as it travels down the septal surface, spreads normally throughout the His-Purkinje system. This explains the initial widening of the paced QRS, expressing initial activation of the septal myocardium (simulating a delta wave) followed by the rapid development of a QRS complex similar to natural waves, which indicates activation of the specific system and LV capture through the Purkinje network.^31^

As in our study, Bonomini et al.^22,32^ found a correlation between PHP, homogeneous activation, cardiac synchronization, and QRS spatial variance analysis (imeSI). According to Bonomini et al., developers of Synchromax® (EXO, Buenos Aires, Argentina), through analysis of variables such as the direction of the electrical impulse (from base to apex or vice versa), QRS duration, and the volume and point-to-point symmetry of the curves obtained between leads D2 and V6, the maximum activation time and the delays in electrical propagation in each ventricular chamber can be determined through mathematical processing^22^(Figure 2A). The imeSI is produced through QRS spatial variance algorithm analysis, with values from 0.0 to 1.0 (00 being perfect synchrony and 1.00 being complete dyssynchrony)^12^(Figure 2B). The D2 lead represents the activity of the interventricular septum, including the direction and velocity of conduction of the electrical stimulus. Likewise, the V6 lead represents activation of the LV free wall. In the absence of conduction disturbances, the D2 lead is positive and has preserved duration, showing physiological activation and conduction time from the base of the IV septum to the apex of the heart. On the other hand, it is expected that V6, when representing the LV, is positive and has a duration and spatial volume similar to D2, since it follows the same activation pattern. Graphically, if there is synchronization, the resulting curves will ideally overlap (D2 and V6 will be identical and homogeneous). The only explanation for these simultaneous and symmetrical ventricular activation curves would be the recruitment of the intrinsic conduction system and coordinated and homogeneous ventricular contraction (synchrony).

A randomized, double-blind, cross-over study^33^ showed that PHP preserves LVEF and mechanical synchrony similarly to RV myocardial septal pacing in patients with high-grade atrioventricular block, narrow QRS, and LVEF < 0.40. Kronborg et al. concluded that in these selected patients, significant ventricular remodeling or heart failure due to PHP is not expected.^33^ In the same way, another randomized study compared 6 months of PHP with 6 months of RV apical pacing in 16 patients with chronic atrial fibrillation and atrioventricular node ablation. Patients undergoing PHP had reduced IV dyssynchrony, improved functional class, significantly improved 6-minute walk test performance, and decreased mitral and tricuspid regurgitation.^34^

### PHP and NS-HBP: similar cardiac synchrony results

Nature designed the cardiac conduction system to activate the ventricles from the endocardium to the epicardium, from the base to the apex, and from right to left. This order is considered the “physiological axis”.^13^ Simultaneous, homogeneous, coordinated, and symmetrical contraction denotes “synchrony.” Our results confirm that His-bundle capture (DDD-His group) results in synchronous ventricular contraction identical to the intrinsic rhythm, which reinforces His-bundle pacing as the gold standard. Likewise, QRS variation analysis confirmed ventricular synchrony with an imeSI < 0.4 for all DDD-His patients except one (imeSI > 0.7), which was due to microdisplacement of the lead from the His position, resulting in capture of the adjacent septal myocardium – one possible complication of this technique.^10^ When myocardial activation occurs, as in “conventional pacing” of the RV (ie, apical and septal muscle alone), electrical conduction takes place through nonspecific tissue (myocardial capture) and outside the specialized His-Purkinje system.^2,28^ This unwanted pacing modality has a characteristic ECG pattern,^26^ and the mechanical result is a loss of efficacy in ventricular contractility. As previously described, the magnitude of dyssynchrony is analytically demonstrated by imeSI as it approaches a value of 1.

Our most important result is that all patients in the DDD-Var group, who began in dyssynchrony (imeSI > 0.7 to 1), recovered homogeneous activation (synchrony) of the ventricles after PHP (92.1% with an imeSI < 0.4; the remainder with an imeSI from 0.4 to 0.69; the median imeSI variation in the DDD-Var group was -0.74). Further reinforcing the similarity of PHP and NS-HBP, according to the criteria of Mala et al.,^25^ the ventricular activation in both groups was in the “physiological” or “probably physiological” axis.

### Paced QRS duration

Proper cardiac functioning depends on a highly coordinated (synchronized) electromechanical system. Conduction abnormalities, such as those caused by purely myocardial pacing, lead to dyssynchrony, which can have deleterious effects (pacing-associated cardiomyopathy, or artificially induzed myocardial dyssynchrony).^3,4,35^ The PHP applied in this study, despite correcting the dyssynchrony in practically all patients, does so at the expense of a significant widening of the QRS complex (Figure 8). Nevertheless, no case exceeded the critical value of 150 ms.^19,28^ Zhang et al.^9^ assessed the acute effect of S-HBP, NS-HBP, and myocardial pacing of the right IV septum on the electrical and mechanical synchronization of the LV, finding that both S-HBP and NS-HBP could restore physiological contraction and mechanical synchrony. This confirms that any modality of HBP can maintain native ventricular activation through the intrinsic conduction system, which is demonstrably more physiological and characterized by better indicators of cardiac synchronization than patients with purely myocardial pacing.^9^ However, in several studies, QRS duration on 12-lead ECG has been used as an indirect marker of electrical synchrony, while prolonged intrinsic or paced QRS duration has been associated with an increased risk of heart failure.^8,36^ In our study, DDD-His was superior to DDD-Var (PHP) in terms of shorter duration of the paced QRS, but in both techniques, a significant widening was noticed which was of the same magnitude. This result was also present in the study conducted by Zhang et al.^9^ during low-output pacing with NS-HBP, in which, despite demonstrating better electrical and mechanical synchrony than myocardial septal pacing, the paced QRS duration was wider than the intrinsic QRS duration.

However, we found that the magnitude of QRS variation before and after the procedure between the groups was not significant (p = 0.436), and that NS-HBP (DDD-His group) clearly captured the native conduction system. This could be explained by the fact that when pacing near but not directly on the His bundle (indirect physiological pacing), fused QRS complexes are produced. An initial enlargement (pseudo-delta wave) is observed, attributable to the concomitant capture of muscle tissue near the His bundle, resulting in 2 depolarization fronts that merge. One front recruits the intrinsic system and activates the LV through the native left branch, while the other briefly travels through the adjacent interventricular septum to the His bundle until it finds and activates the conduction system through the right side, similar to what is observed in pre-excitation syndromes with accessory para-Hisian pathways.^31^ However, although mechanical dyssynchrony is a frequent finding in patients with wide QRS complexes, QRS width alone does not appear to be an efficient marker for diagnosing dyssynchrony.^12,13,22^ The activation axis and the morphological dispersion of ventricular depolarization would be more striking features, as shown by Bonomini et al.^22^ This important paradigm shift was confirmed in a study that compared QRS compared QRS spatial variance analysis method with echocardiography and found that ECG analysis through spatial variance has better sensitivity and negative predictive value to detect mechanical dyssynchrony than QRS duration or conventional ECG alone.^32^ In our study, DDD-His performed better compared to DDD-Var (PHP) in terms of paced QRS duration. However, in both techniques, despite the noticed QRS widening, synchrony was achieved in the same magnitude suggesting that QRS coordination (synchrony) is more important than QRS duration.

### Physiological pacing with both techniques?

Recent publications^26,27,37^ have described how to distinguish between purely myocardial activation and direct or indirect capture of the intrinsic conduction system in ECG (Figure 3). Analysis of these criteria (performed in the present study) would reduce the risk of incorrectly identifying nonspecific myocardial capture as PHP which, in some cases, is associated with clinical results similar to conventional RV pacing.^26,28^ When electrical impulses are conducted through non-specialized muscle tissue, the IV septum is abnormally activated, which leads to a marked delay in LV lateral wall activation, causing morphometric changes and QRS distortion. After exhaustive application of these electrocardiographic principles^26,27^ we found no significant difference (p = 0.999) between the groups. Of note, in the DDD-Var group, the absence of notching in the V1 lead and a time < 100 ms between the stimulus (spike) and RWPT in V6 were the most striking characteristics of physiological pacing, being similar in both PHP and NS-HBP. On the other hand, in the DDD-His group, perhaps corroborating the NS-HBP results, the variable most associated with non-physiological pacing was RWPT > 100 ms. However, this can be explained by the interval from the stimulus until penetration and capture of the His bundle. The QRS variation analysis method (Synchromax®) consistently supports this synchrony, which is comparable to NS-HBP, with an imeSI < 0.4 with overlapping activation curves.

### Safety and efficacy of PHP

Due previously described nuances of the His-bundle pacing technique, we preferred to apply this strategy to patients with sinus node dysfunction and intact atrioventricular conduction. Patients with intra- and infra-His blocks present additional challenges to this approach and often require guaranteed RV pacing by a second backup lead (greater resource consumption and greater risk of complications).

Positioning the RV lead in the uppermost proximal regions of the interventricular septum is simpler, more easily reproduces para-Hisian activation, and is feasible for any service that performs pacemaker implants with radiological anatomy.^19^ Our results confirm that PHP is viable and especially safe for pacing-dependent patients, in whom pacing by His capture can be more challenging.^10^ Furthermore, PHP has a shorter learning curve and a significantly shorter exposure to intraoperative fluoroscopy (Figure 9).

It should also be pointed out that pacemaker programming and resolving intraoperative problems related to direct HBP can be an obstacle.^37^ On the other hand, favorable electronic parameters, such as R waves of significantly better amplitude, were found in the DDD-Var group (Figure 10). Thus, PHP efficaciously and safely overcomes some of the classic inconveniences of physiological pacing compared to HBP. Hanifin et al.^37^ suggest that operators who perform HBP need specific training to resolve adversities both intra-procedurally and during programming adjustments. This scenario naturally increases the consumption of health resources.^37^

Low R wave amplitude, an unwanted occurrence during HBP, can lead to problems with intrinsic electrical activity detection, resulting in pacemaker dysfunction and programming conflicts.^37^ It should also be pointed out that direct Hisian capture usually requires higher output energy (voltage), which results in shorter generator battery life.^10^ In our study, an exhaustive search for adequate pacing parameters may have resulted in a higher fluoroscopy time for this group.

### Review of physiological pacing classification: direct vs. indirect

This pioneering study compared LV electromechanical synchrony through instantaneous processing of QRS spatial variance in patients undergoing NS-HBP or PHP. Bearing in mind that a lack of difference does not strictly indicate equivalence, based on our findings we propose a reclassification of physiological pacing based on the degree of “direct” or “indirect” involvement (capture) of the His-Purkinje system. Direct physiological pacing would combine “rigorous capture” through mapping of the intrinsic electrical system, demonstrating recruitment of the His bundle (S-HBP) or one of its branches (left branch pacing - deep septal technique). Indirect physiological pacing would be represented by NS-HBP and PHP, with proof of rapid and homogeneous (synchronous) ventricular activation. However, it would involve brief, partial, and variable capture of the Hisian region of the myocardium (resulting in a pseudo-delta on the ECG). Indirect physiological pacing would include the type II intraseptal anatomical variant of His,^24,37,40,41^ which can include more than 30% of cases and almost always produces NS-HBP.

Finally, must be recognized that direct pacing of the bundel of His is the gold gold standard for preserving a physiological activation pattern. Non-selective indirect forms and PHP are variants that, as shown in this study, could preserve ventricular contractile synchrony, avoiding the potential deleterious effects of “conventional pacing”.^9,12^

PHP-type indirect physiological pacing can recruit the intrinsic conduction system.^31^ This interesting and promising pacing modality, when combined with tools such as QRS spatial variance analysis (imeSI - Synchromax®), makes the method more easily reproducible and effective.

### Limitations

This study’s main limitations were that it included a relatively small series of patients with heterogeneous indications for pacemaker implantation, even though the synchrony analysis was performed with overstimulation and uniform ventricular capture (VVI mode). Moreover, it was a single-center study with limited retrospective data analysis. It should also be considered that, despite the specific methodological conditions, synchrony was assessed by an indirect method, not considering other variables that can alter cardiac electrical conduction. Finally, although the long-term effects of maintaining synchronization by avoiding cardiomyopathy through pacing could be determined with longer follow-up, this was not the objective of the present study. The focus and strength of this study lies in its comparison of physiological pacing strategies during the perioperative implantation process.

## Conclusion

We found that both PHP and NS-HBP can result in similar cardiac synchronization, which places both in a new classification: indirect physiological pacing. Although this strategy is promising and attractive, a valid and comparable alternative when carried out with methodological rigor, both the electrocardiographic analysis of QRS spatial variance and indirect evidence of conduction system capture must be validated, as any new technology or procedure, in new studies with a larger number of patients.
